# A multiplex PCR assay for the detection of five influenza viruses using a dual priming oligonucleotide system

**DOI:** 10.1186/s12879-015-0818-y

**Published:** 2015-02-25

**Authors:** Xuezheng Ma, Huanzhou Xu, Lei Shi, Pengfei Yang, Liping Zhang, Xiaohong Sun, Wei Zhen, Kongxin Hu

**Affiliations:** Institute of Health and Quarantine, Chinese Academy of Inspection and Quarantine, |No.A3, Gaobeidian North Road, Chaoyang District Beijing, 100123 China; Department of Disease Control and Prevention, Shenzhen International Travel Health Care Center, Shenzhen, Guangdong Province 518045 China; Huaian Center for Disease Control and Prevention, No.118, Huaihai North Road, Qinghe District, Huaian, Jiangsu Province China

**Keywords:** Dual priming oligonucleotide, DPO, Multiplex PCR, Influenza

## Abstract

**Background:**

A cost-effective, accurate and rapid simultaneous multiplex assay is required for testing and diagnoses of conventional and emerging viruses in clinical virology laboratories. We developed and optimized a dual priming oligonucleotide (DPO) multiplex PCR assay for detecting influenza viruses including seasonal H1N1, 2009 pandemic H1N1, H3N2, influenza B and H5N1.

**Methods:**

The optimized multiplex DPO PCR was used to detect 233 clinical human samples. The results were compared to those obtained with RT-qPCR, conventional PCR and immunochromatographic assay.

**Results:**

Specificity analysis revealed that the DPO PCR assay amplified each target virus without any cross-amplification. Statistical analysis demonstrated that the multiplex DPO-PCR sensitivity was higher than for the immunochromatographic assay and lower than for qPCR, while no significant difference was observed compared with conventional PCR, when detecting influenza A and B. Additional experiments using the same sample panel indicated no significant differences between the number of positive samples detected by multiplex DPO PCR and RT-qPCR when applying a Cq with a value lower than 30.

**Conclusions:**

The five-targeted simultaneous multiplex DPO PCR assay could be easily adopted into routine practice. This approach is cost effective with a short running time, low technical requirements for the detection of influenza virus and early diagnosis in clinical laboratories.

## Background

Influenza viruses are the major pathogens causing respiratory diseases with severe morbidity and mortality worldwide [1]. Novel and recombinant strains of influenza viruses have caused pandemics resulting in millions of deaths. The rapid detection of these viruses is essential for a medical response and infection control [1]. A low-cost and rapid identification of influenza types and subtypes in clinical patients is essential for initial clinical treatment and avoidance of antibiotic misuse, as well as prevention of influenza virus transmission, especially in resource-limited regions [1].

Molecular assays are highly sensitive and specific for detecting influenza viruses such as rapid immune colloidal gold diagnostic tests, immunofluorescence, enzyme-linked immunosorbent assay (ELISA) and viral culture. Nucleic acid based tests for respiratory viruses are currently used as a rapid and sensitive diagnostic approach for clinical specimens. A cost-effective, accurate and rapid multiplex assay is highly desirable in clinical virology laboratories for testing and diagnosing common and emerging viruses. Even though the multiplex real time polymerase chain reaction (PCR) is a rapid and sensitive method for the detection of respiratory viruses, such assays are limited to a maximum of four multiplexed targets, and the high cost of reagents and equipment involved is particularly limiting for laboratories with low financial constraints [2].

Therefore, a novel dual priming oligonucleotide (DPO) technology was developed to solve these problems. The DPO primer contains two separate priming regions joined by a polydeoxyinosine linker, which assumes a bubble-like structure not involved in priming, but which delineates the boundary between two regions of the primer [3]. This structure confers distinct annealing properties on the two primer segments. The longer 5′-segment initiates stable priming, while the short 3′-segment determines target-specific extension [3]. The advantage of DPO based PCR is the blocking extension of non-specifically primed templates under less than optimal PCR conditions [3]. DPO technology was commercialized and used in many recent studies to test different respiratory viruses [3]. Additionally, several studies have compared DPO based multiplex PCR with other rapid methods for the detection of influenza viruses. The results suggested that the DPO based multiplex PCR system had a higher sensitivity and specificity than the other methods tested [4-7]. However, most of these recent studies, including those using commercial DPO based rapid detection kits, tested numerous different respiratory viruses or different types of influenza viruses. Only one commercial kit (Seeplex® Influenza A/B OneStep Typing, Seegene, Seoul, Korea) can simultaneously detect influenza A, B and subtypes of influenza A including 2009 pandemic H1 and Human Seasonal Influenza A (H1 and H3). Recently, the live poultry market has posed a high risk for human infection following the avian infection in Beijing, China [8]. The highly pathogenic avian influenza H5N1 was identified in local poultry and wild birds is enzootic in China [8]. Therefore, it is important to develop a rapid and simple method to detect avian influenza viruses. To our knowledge, no previous study has reported the development of a DPO multiplex assay for the simultaneous detection of influenza A, B and subtypes of influenza A including H5N1.

The purpose of this study was to develop and optimize a DPO multiplex PCR assay for detection of influenza A and B viruses, including the influenza A subtypes, such as seasonal H1N1, 2009 pandemic H1N1, H3N2, and avian influenza H5N1. Additionally, the study compared and evaluated DPO PCR, qPCR, conventional PCR and immunochromatographic assay for the detection of influenza viruses in clinical specimens.

## Methods

### Clinical samples

This study randomly screened 233 clinical specimens from feverish patients including human oropharyngeal swabs, nasopharyngeal swabs and sputum from Chinese Academy of Inspection and Quarantine, Center for Disease Control of China, Beijing, China and the Shenzhen International Travel Health Care Center. Two H5N1 specimens were obtained from avian species from the Center for Disease Control of China. Additionally, 59 respiratory samples including parainfluenza viruses 1/2/4 (PIV1/2/4), human rhinovirus (HRV), human metapneumovirus (hMPV), adenovirus (ADV), coronavirus 229E (CoV229E), respiratory syncytial virus A/B (RSV A/B) and bocavirus (BoV) were collected to assess the specificity of the multiplex DPO assay.

### DPO primer design

DPO is a primer system for PCR that contains a bubble-like polydeoxyinosine linker that separates a single primer into two unequal regions. The five influenza virus sequences were aligned by MEGA5.1 [9]. Five pairs of DPO primers were designed based on the specific regions for each virus (Table 1). The optimal numbers of polydeoxyinosine for each DPO primer was determined by testing 3–8 poly (I) linkers for each target virus. The amplified products were analyzed by 2.0% agarose (Biowest, Hongkong, China) gel electrophoresis.

**Table 1:** **DPO primers used for multiplex DPO PCR**

**Virus**	**Target gene**	**Primer sequence (5′–3′)**	**Amplicon size (bp)**
H1N1 pdm09	HA	F:TAGTGCTGACCAACAAAGTCTCTAIIIIIATGCAG	231
		R:GCATTTCTTTCCATTGCGAATGCIIIIITCGGTAC	
sH1N1	HA	F:TGCGAATYACTGATTTCCAAGGAIIIIIGGTCCTA	193
		R: CTCCGGTTACRGTGTGGTGGGGIIIIIAGCTCTCT	
H3N2	HA	F:ACGCTGTGCCTTGGGCACCATGCAIIIIIAAACGG	537
		R:GTCMTTGTCCGTACCCGGGTGGTGIIIIICCCAAA	
H5N1	HA	F:GAGAGATTGTAGTGTAGCTGGATIIIIICTCGGAA	328
		R:CTTTATTGTTGGGTATGTRCTGTTIIIIITGATAAGCC	
FluB	PB1	F:TTGGCTATGACTGAAAGAATAACCAIIIIIAGCCCA	401
		R:GCATTAACAAATAGAGCAAAATCATIIIIIGATTGC	

HA: hemagglutinin gene; PB1:polymerase basic 1 gene; F: forward primer; R: reverse primer; bp: base pair.

### Viral RNA extraction and cDNA synthesis

All clinical samples were concentrated and enriched by a virus concentration device to increase the virus load. Viral RNA was extracted from viral solutions using the QIAamp Viral RNA Mini Kit (Qiagen, Germany) following the manufacturer’s specifications. A volume of 140 μl of virus solution was mixed with 560 μl buffer AVL-carrier RNA and incubated for 10 min at room temperature. After adding 560 μl ethanol (100%), the solution was mixed thoroughly by pulse-vortexing and then transferred to a spin-column. After a series of washing and drying steps, 60 μl of RNase-free water was used to elute RNA, which was stored at −70°C. The master mix for reverse transcription was prepared using a reverse transcription system (Promega, USA) under the following conditions: a mixture of 5 μl RNA template, 1 μl Random primer and 2 μl RNase-free water was denatured at 95°C for 2 min and cooled on ice for 2 min. Then 4 μl of MgCl_2_ (25 mM), 2 μl dNTPs (10 mM), 2 μl reverse transcription 10× buffer, 30 U of AMV reverse transcriptase, 25 U of RNasin ribonuclease inhibitor and nuclease-free water were added to a final volume of 20 μl. Subsequently, the mixture was incubated at 42°C for 90 min and 72°C for 10 min. Finally, the cDNA was stored at −20°C.

### Multiplex DPO PCR protocol

Multiplex DPO PCR reactions contained 1.5 U of Takara Taq (Takara Bio, Dalian, China), 2.5 μl of 10× PCR buffer, 2.0 mM Mg^2+^ and 250 μM dNTPs. The optimal concentration of DPO primer mix are listed in Table 2. 2009 pandemic H1N1, seasonal H1N1, H3N2 and H5N1 primers at a concentration of 0.8 μM and influenza B primers at a concentration of 1.6 μM, 2 μl cDNA and nuclease-free water were added to a total volume of 25 μl. The amplification conditions of the multiplex PCR were as follows: pre-denaturation step for 5 min at 94°C, 40 cycles of denaturation at 94°C for 30 s, annealing at 60°C for 30 s, extension at 72°C for 1 min, followed by a final extension step at 72°C for 10 min. The multiplex DPO PCR was performed with an Applied Biosystems® 2720 Thermal Cycler (Life Technologies, NY, USA). The amplified products were analyzed by 2.0% agarose gel electrophoresis.

**Table 2:** **Optimal DPO primer mix concentrations**

**Component**	**H1N1 pmd09**	**Seasonal H1N1**	**H3N2**	**H5N1**	**Influenza B**
Forward Primer (μM)	20	20	20	20	40
Reverse Primer (μM)	20	20	20	20	40
25× primer mix (μM)	0.8	0.8	0.8	0.8	1.6

### Analytical sensitivity and specificity

The partial HA and PB1 gene sequences of each virus (seasonal H1N1, CY082460, nt 250–442; 2009 pandemic H1N1, CY087016, nt 616–846; H3N2, CY091581, nt 96–632; H5N1, AB598119, nt 210–537; influenza B, CY069569, nt 955–1355) were synthesized and inserted into pGM-T (Tiangen, China) to construct five specific plasmids. These plasmids were then *in vitro* transcribed using the RiboMax™ Large Scale RNA Production System-T7 according to the manufacturer’s instructions (Promega, USA). The RNA concentration were detected by spectrophotometer (NanoDrop, Delaware, USA) and then reverse-transcribed to cDNA as previous instruction (Promega, USA). The initial concentration of sH1N1, H1N1pdm09, H3N2, H5N1 and FluB were 1.3 × 10^11^ copies/ml, 5.7 × 10^10^ copies/ml, 6.2 × 10^10^ copies/ml, 1.3 × 10^10^ copies/ml and 3.5 × 10^10^ copies/ml, respectively.

A ten-fold serial dilution of plasmid DNA was used to compare the sensitivity levels of the multiplex PCR and the single conventional PCR for amplifying each type of influenza. Then, a pooled solution of all five templates was diluted in series (10^7^–10^1^ copies/ml), and detected by multiplex DPO primers to determine the sensitivity. The specificity test comprised the five pooled templates amplified by each pair of DPO primers individually. Additionally, the measurement of one-step multiplex DPO PCR specificity was determined using eight respiratory viral RNA preparations: PIV1/2/4, HRV, hMPV, ADV, CoV229E, RSVA, RSVB and BoV.

### Sequencing

The amplified conventional PCR products were sequenced to evaluate the specificity of the assay. Sequencing was performed using an ABI PRISM 3730 DNA Sequencer and sequences obtained were confirmed by the GenBank (National Center for Biotechnology Information) database with the Basic Local Alignment Search Tool (BLAST).

### RT-qPCR

Total RNA from 140 μl clinical samples was separately extracted using QIAamp Viral RNA Mini kit (Qiagen, Valencia, CA, USA). RNA was eluted in 60 μl of elution buffer and stored at −80°C. The singleplex RT-qPCR was performed using an Applied Biosystems® 7500 Real-Time PCR System (Applied Biosystems® by Life Technologies, NY, USA) to test all clinical samples. The primers and probes used for the RT-qPCR were suggested by WHO Information for Molecular Diagnosis of Influenza Virus in Humans - update (November 2012) [10]. RT-qPCR was performed with an AgPath-ID™ One-Step RT-PCR Kit (Ambion, Applied Biosystems® by Life Technologies, NY, USA). A total of 25 μl RT-qPCR mixture included 12.5 μl of 2× RT-PCR buffer, 1 μl 25× RT-PCR enzyme mix, 0.5 μl one-step RT-PCR master kit (Qiagen), 0.5 μl (20 μM) of each primer, 0.3 μl (10 μM) of probe, 4.7 μl of nuclease-free water and 5 μl of extracted RNA. The thermocycling parameters were as follows: reverse transcription (RT) at 45°C for 10 min, RT inactivation at 95°C for 10 min and fluorescence detection for 40 cycles at 95°C for 15 sec and annealing at 60°C for 45 sec. RT-qPCR data was analyzed by SDS software from Applied Biosystems®. Amplification curves were evaluated by the threshold line being placed above the background signal, intersecting the initial exponential phase of the curve. Amplification of influenza virus was observed at a quantification cycle (Cq) value of 35. A test result was considered positive when a well-defined curve that crossed the threshold cycle within 35 cycles was observed.

### Conventional PCR protocol

The detection kit used in our study was from Takara Biotechnology (Dalian) Co., Ltd. A 25 μl reaction system was set up containing 2 μl template RNA, 0.125 μl Takara Taq (250 U/μl), 0.5 μl of each primer (10 μM), 2 μl of 10× PCR Buffer (Mg2+ Plus), dNTP mixture (2.5 mM) and 17.4 μl RNase free water. The test was performed using a Applied Biosystems® 2720 Thermal Cycler. The PCR reaction was amplified under the following conditions: pre-denaturation step for 5 min at 94°C, 40 cycles of denaturation at 94°C for 30 s, annealing at 60°C for 30 s, extension at 72°C for 1 min, followed by a final extension step at 72°C for 10 min. The PCR was performed by Applied Biosystems® 2720 Thermal Cycler. The amplified products were analyzed by 2.0% agarose gel electrophoresis.

### Alere BinaxNOW® Influenza A&B Card rapid detection assay

The colloidal gold immunochromatographic assay was applied for the detection of influenza A and B. The procedure followed the manufacturer’s instructions.

### Comparison of multiplex DPO, conventional and RT-qPCR, and Alere BinaxNOW® Influenza A&B Card rapid detection assay for influenza A and B

All clinical specimens were screened for seasonal H1N1, 2009 pandemic H1N1, H3N2, influenza B and avian influenza H5N1 by multiplex DPO PCR. RT-qPCR and conventional PCR for the detection of influenza A and B were used to validate the multiplex DPO PCR method and results were compared to those obtained by immunochromatographic assay.

### Statistical analysis

Statistical analysis was performed by SPSS18.0 (IBM, New York, USA). The chi-squared test was used to analyze data and a *P* value of < 0.05 was considered statistically significant.

### Ethical statement

The study was conducted according to the protocol approved by the Human Research Ethics Committee, Chinese Academy of Inspection and Quarantine in compliance with the provisions for human research in the Helsinki Declaration (ES-0823696/2014/376HQ). Written informed consent was obtained from all the participants.

## Results

### Optimized numbers of poly I linkers in DPO primer design and annealing temperature for multiplex DPO PCR

**Figure 1:**
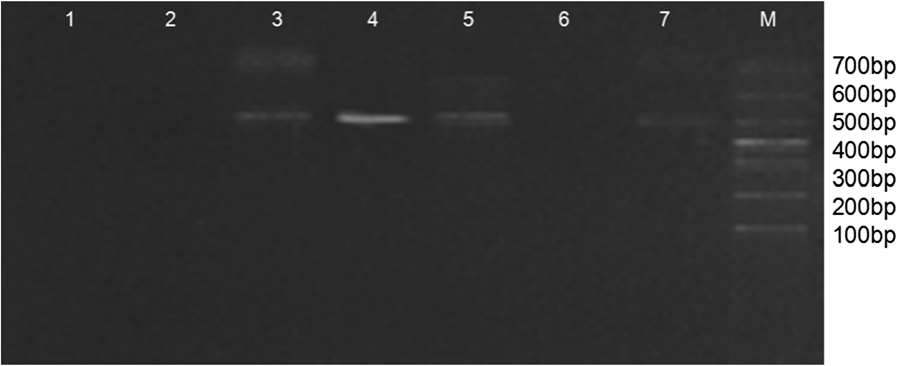
**Optimized number of poly (I) linkers in DPO primer design for sH1N1, H1N1pdm09, H3N2, H5N1 and FluB by multiplex DPO PCR.** Lane 1: negative control; Lane 2–7: three to eight polydeoxyinosine (I) linkers in DPO primers tested for each target virus; Lane M: DNA marker.

**Figure 2:**
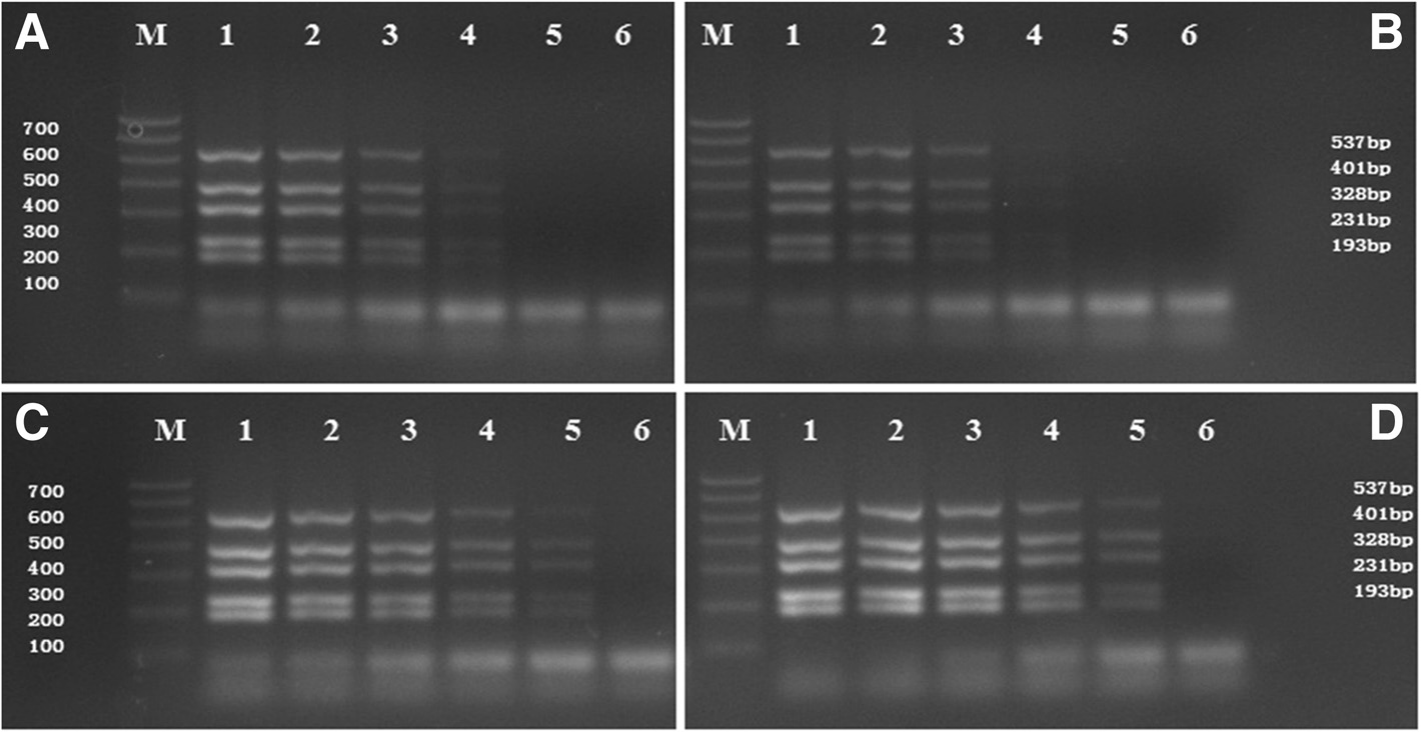
**Optimized annealing temperature for sH1N1, H1N1pdm09, H3N2, H5N1 and FluB by multiplex DPO PCR.** (**A**: *Tm* = 46°C; **B**: *Tm* = 50°C; **C**: *Tm* = 55°C; **D**: *Tm* = 60°C). Lanes: M: DNA Marker; 1–6: 10^7^–10^2^ viral particles/μl. 537 bp for H3N2, 401 bp for FluB, 328 bp for H5N1, 231 bp for H1N1pdm09, and 193 bp for sH1N1.

The number of poly (I) in DPO primers impacts both the sensitivity and specificity of the DPO PCR amplification. This study therefore tested various numbers of poly (I) linkers, with five linkers proving optimal (Figure 1). Because the influenza viral gene are highly mutable, four annealing temperatures (*Tm*) were selected and applied to different concentrations of the mixed templates. When the *Tm* was 46°C and 50°C, the lowest template concentration was 10^4^ viral particles/ml (Figure 2). When the *Tm* was increased to 55°C, faint bands (10^3^ viral particles/ml) were observed. When the *Tm* reached to 60°C, distinct bands indicated the lowest concentration of mixed template was 10^3^ viral particles/ml for multiplex DPO PCR.

### Multiplex DPO PCR sensitivity

**Figure 3:**
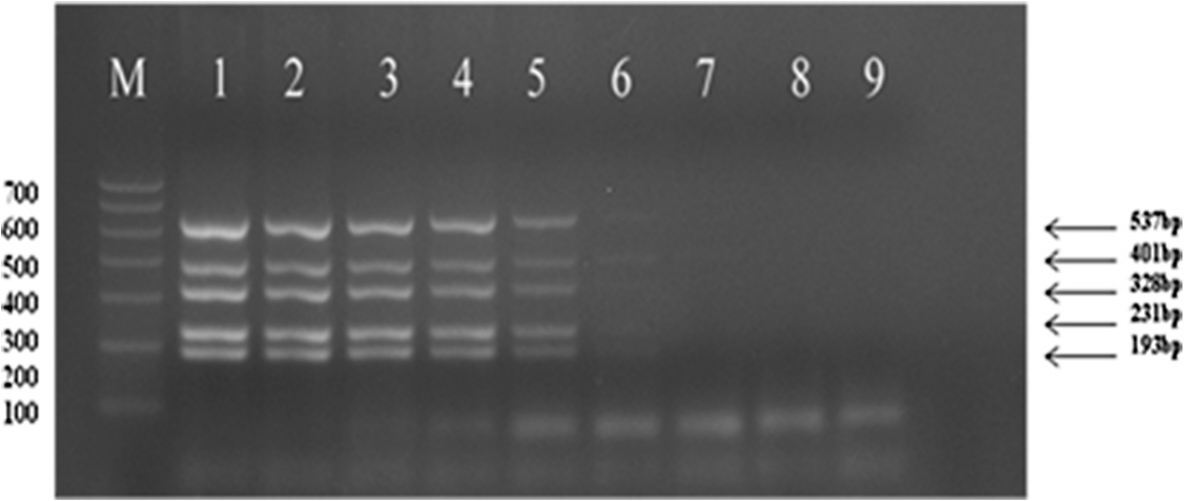
**Detection sensitivity of sH1N1, H1N1pdm09, H3N2, H5N1 and FluB by multiplex DPO PCR.** Lanes: M: DNA Marker; 1–8: 10^7^–10^1^ viral particles/ml; 9: negative control. 537 bp for H3N2, 401 bp for FluB, 328 bp for H5N1, 231 bp for H1N1pdm09, and 193 bp for sH1N1.

A 10-fold dilution series was used to determine the lowest concentration detected by the multiplex DPO PCR. This was found to be 10^3^ copies/ml for sH1N1, H1N1pdm09, H3N2, H5N1 and FluB; although very faint bands were also observed in lane 6 for 10^2^ viral particles/ml (Figure 3). The sizes of the amplified product were detected by 2% agarose gel, and determined to be 537 bp for H3N2, 401 bp for FluB, 328 bp for H5N1, 231 bp for H1N1pdm09, and 193 bp for sH1N1. Although a pooled mixture of the five virus templates was used for amplification by the multiplex primers, no cross-amplification was observed.

### Multiplex DPO PCR specificity

**Figure 4:**
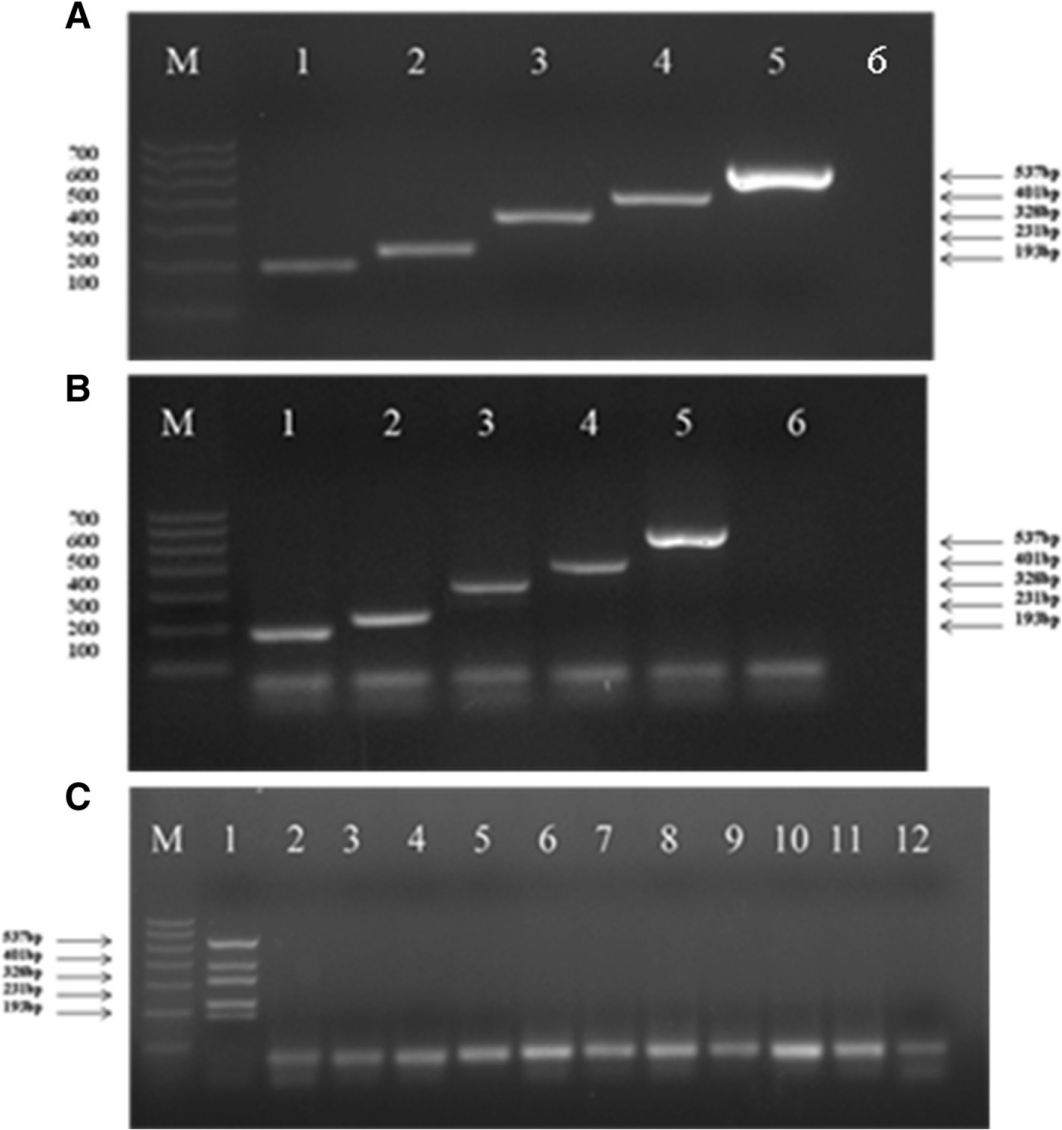
**Detection specificity of sH1N1, H1N1pdm09, H3N2, H5N1 and FluB by multiplex DPO PCR. A**: Each of the five primer pairs was applied individually to amplify a pooled mixture of the five different target cDNAs (Lanes 1–5: sH1N1, H1N1pdm09, H3N2, H5N1 and FluB, respectively). **B**: A pooled mixture of the five primer pairs was applied to each of the five target cDNAs individually for amplification (Lanes 1–6: sH1N1, H1N1pdm09, H5N1, FluB, H3N2 and negative control). **C**: Detection of other respiratory viruses (Lanes 1–12: positive control, PIV1, PIV2, PIV4, HRV, hMPV, ADV, CoV229E, RSVA, RSVB, BoV and negative control).

A single pair of DPO primers was used to amplify sH1N1, H1N1pdm09, H3N2, H5N1 and FluB mixture to test the assay specificity. Each primer set produced a single amplified band without non-specific amplification (Figure 4A). Additionally, the multiplex PCR primers were used to amplify each target virus template. Agarose gel analysis confirmed the multiplex PCR amplification for each lane containing sH1N1, H1N1pdm09, H3N2, H5N1 and FluB, respectively (Figure 4B). No amplification was observed for PIV1/2/4, HRV, hMPV, ADV, CoV229E, RSVA, RSVB or BoV when using the multiplex DPO primers (Figure 4C).

### Screening of clinical specimens by multiplex DPO PCR

**Figure 5:**
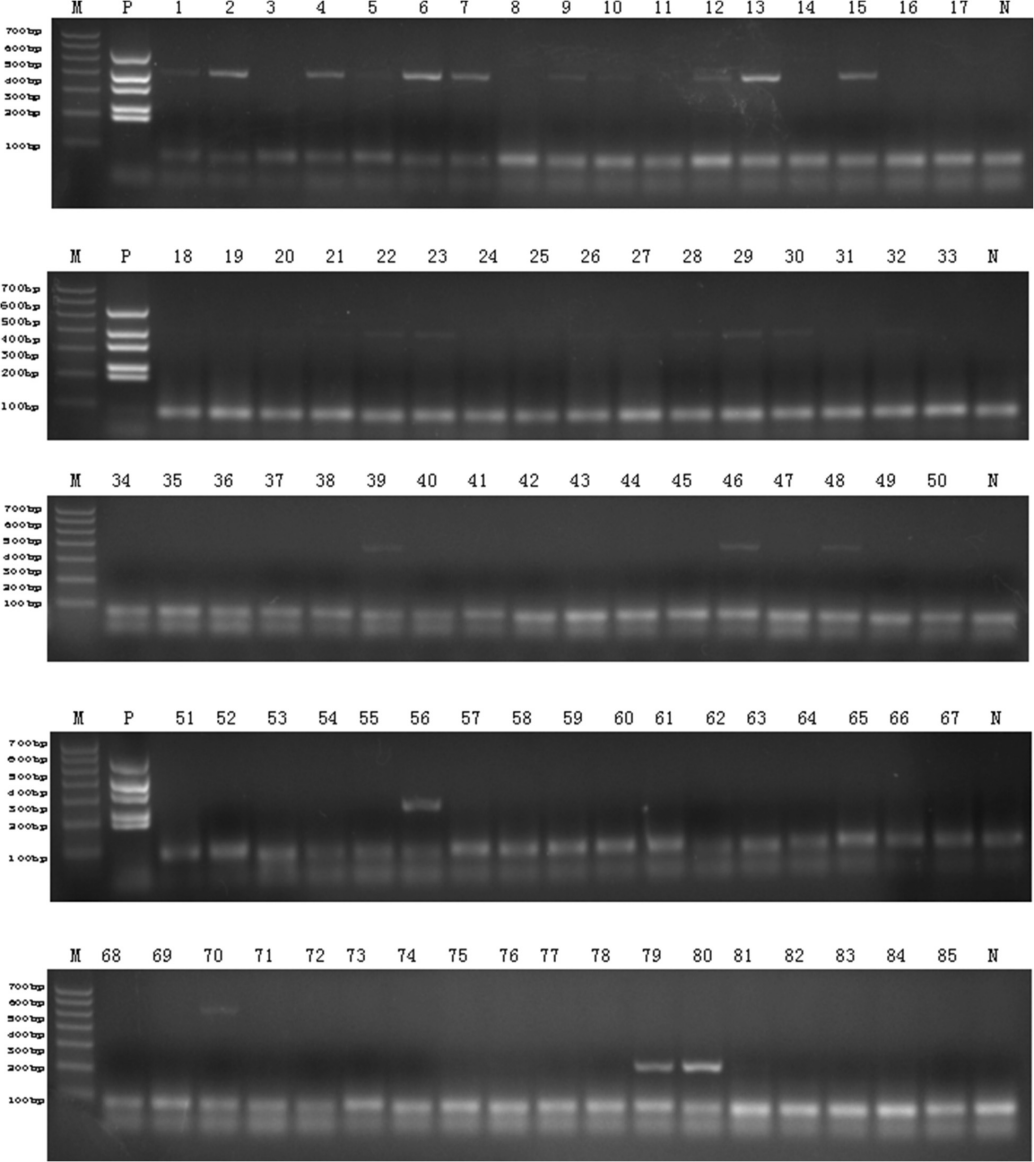
**The selected result of clinical samples by multiplex DPO PCR.** Selected 85 screening result for influenza A and B detection using multiplex DPO PCR. Lane M: DNA marker; lane P: positive control; lane N: negative control.

Using singleplex RT-qPCR as a standard, the overall positives were 153. Of 153 positive specimens, 84 were identified as influenza A and 69 as influenza B. Figure 5 shows the selected multiplex DPO PCR result for clinical sample detection. The percentage of influenza A and B positive samples detected by multiplex DPO PCR in concordance with real time RT-PCR was 66.67% and 62.32%, respectively (Table 3).

**Table 3:** **Clinical specimens tested by qPCR and multiplex DPO PCR**

	**Positive influenza A**				**Positive influenza B**	**Negative samples**
	**Seasonal H1N1**	**2009 pandemic H1N1**	**H3N2**	**H5N1**		
RT-qPCR	14	39	29	2	69	80
Multiplex DPO PCR	13	25	16	2	43	134
Positive Concordant Percentage	56 (66.67%, 56/84)				43(62.32%, 43/69)	____

DPO PCR positive influenza A and B specimens were assessed and confirmed by sequencing to prevent false-positive or false-negative results.

### Comparison of different methods for the detection of influenza A and B

**Figure 6:**
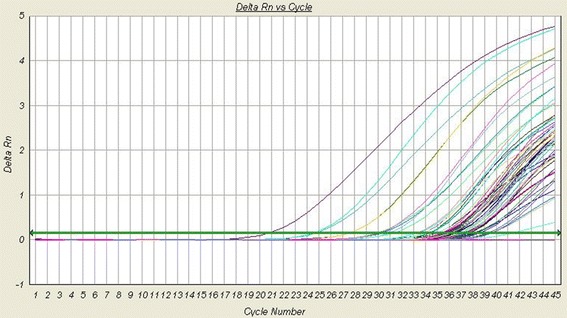
**The selected result of clinical samples by qPCR.** Selected 45 screening results for influenza A and B detection using qPCR.

**Figure 7:**
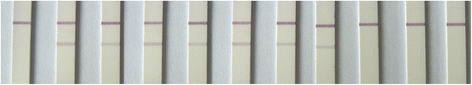
**The selected result of clinical samples by immunochromatographic assay.** Selected result screening for influenza A from 9 clinical samples. P: positive control; N: negative control.

**Figure 8:**
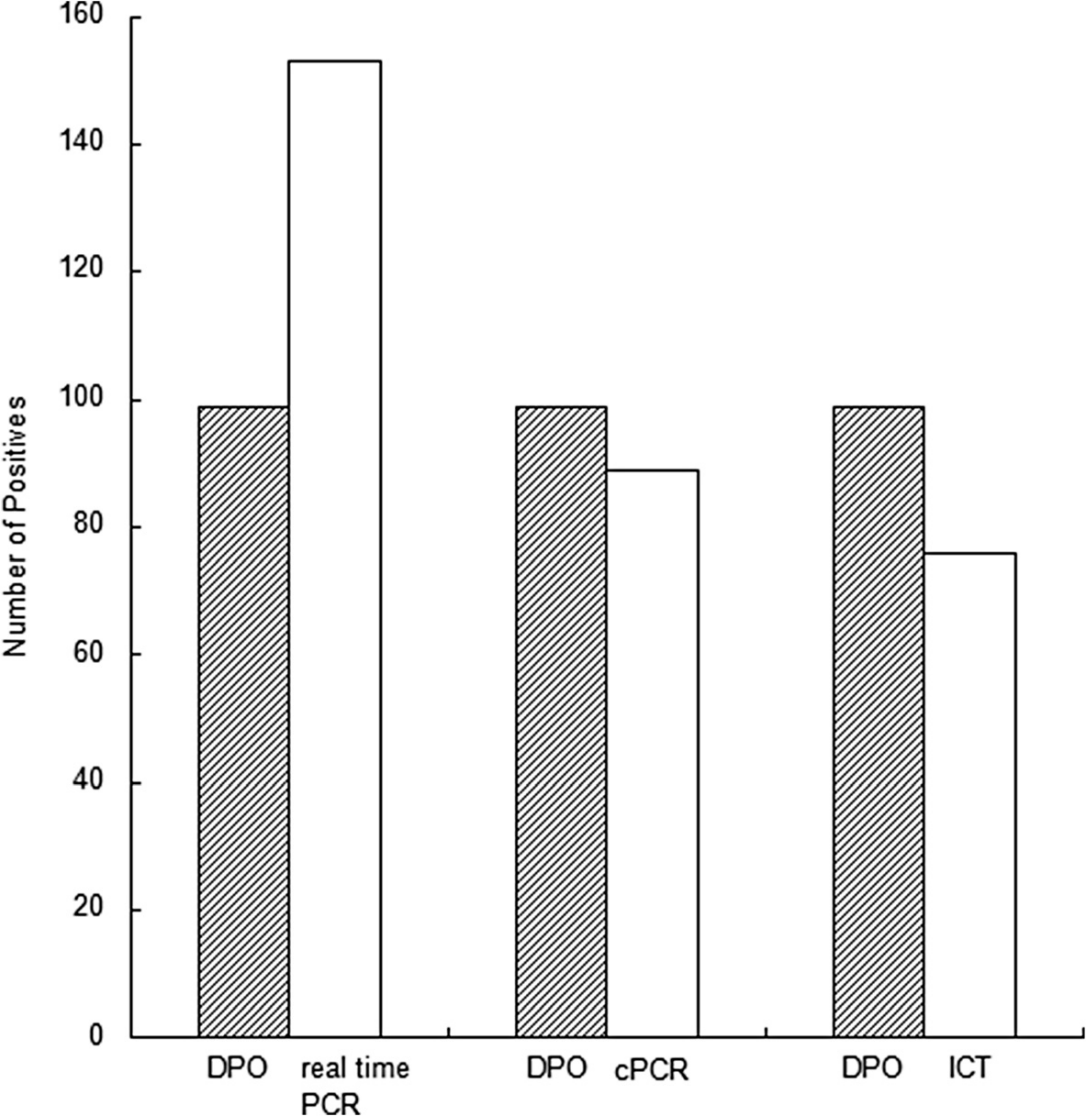
**Comparison of three different PCR methods for all influenza A and B samples.** Shaded bars represent the number of positive samples detected by DPO PCR; clear bars represent real time PCR positive samples, conventional PCR positive samples (cPCR), and positives detected by immunochromatographic test (ICT), respectively.

**Figure 9:**
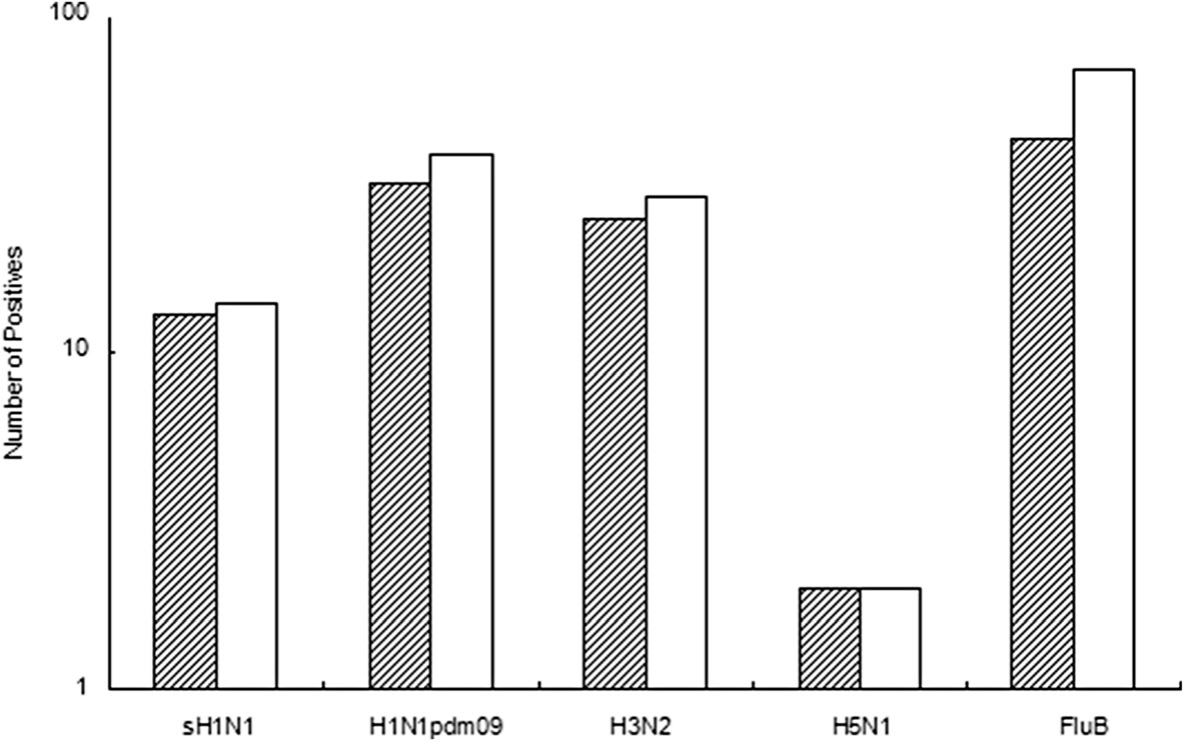
**Comparison of DPO PCR and qPCR positive samples for sH1N1, H1N1pdm09, H3N2, H5N1 and FluB.** Shaded bars represent the number of positive samples detected by DPO PCR; clear bars represent qPCR positive samples.

**Figure 10:**
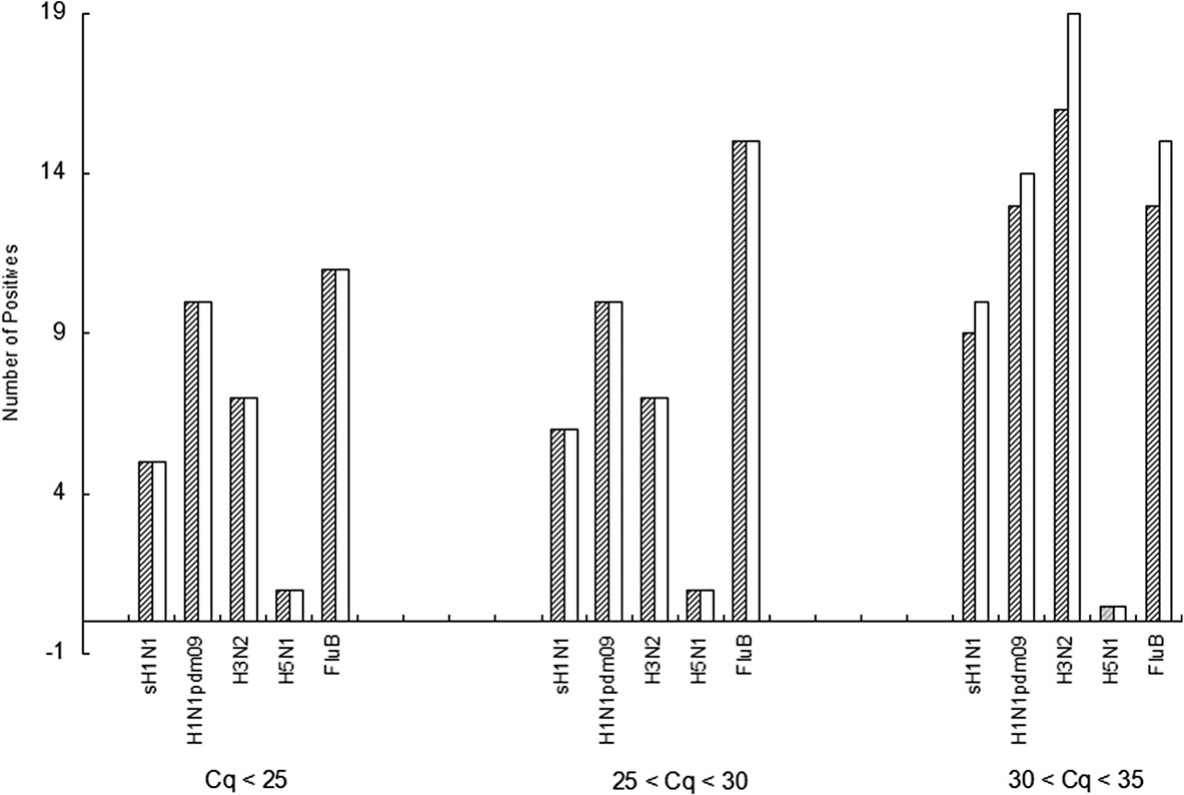
**Comparison of positive results between DPO and qPCR in three different Cq ranges.** Shaded bars represent the number of positive samples detected by DPO PCR; clear bars represent qPCR positive samples.

Multiplex DPO PCR, RT-qPCR, conventional single-target PCR and commercial colloidal gold immunochromatographic assay Alere BinaxNOW® Influenza A&B Card (Alere, CA, USA) were used to detect influenza A and B. The selected test results for qPCR and immunochromatographic assay are shown in Figures 6 and 7, respectively. The order of positive detection efficiency from highest to lowest was RT-qPCR, multiplex DPO PCR, conventional PCR and immunochromatographic assay (Figure 8). The efficacy differences between DPO PCR and qPCR for sH1H1, H1N1pdm09, H3N2, H5N1and FluB are shown in Figure 9. Multiplex DPO PCR displayed 70% correlation with qPCR positive results for influenza A, while influenza B results showed lower correlation with qPCR. qPCR positive results that were inconsistent with multiplex DPO PCR samples were selected for further testing and the expected Cq was recorded. Figure 10 shows that different numbers of positive samples were observed for DPO PCR and RT-qPCR when using three different ranges of Cq values. When Cq was less than 25 or in the range of 25–30, no difference was observed between DPO and qPCR in terms of the number of positive samples detected. When the Cq value was between 30 to 35, the number of positive results obtained by qPCR were greater than those for DPO PCR.

### Statistical analysis of different detection methods

Statistical analysis showed that the sensitivity of the multiplex DPO assay was lower than for qPCR (*P* <0.05), higher than for the colloidal gold immunochromatographic assay (*P* <0.05), but not significantly different from conventional PCR (*P* >0.05) (Figure 8). When the Cq value was lower than 30, no significant difference between multiplex DPO PCR and qPCR when comparing the positive results (Figure 10).

## Discussion

A rapid, accurate and low-cost diagnostic tool for influenza detection is important for the control and prevention of annual influenza pandemics. A previous study described the development of a multiplex DPO PCR assay for the simultaneous and specific detection of influenza A and B [11]. Here, we demonstrated the optimization of one-step multiplex DPO PCR for use as a sensitive and specific method to detect seasonal H1N1, 2009 pandemic H1N1, H3N2, and avian influenza H5N1.

### Optimized annealing temperature for multiplex DPO PCR

For most conventional PCR methods, a lower annealing temperature gives a higher sensitivity, which might increase the nonspecific amplification rate [5,12]. However, if the *Tm* is increased to maintain high specificity, then the test sensitivity will in turn decrease. In this study, when the *Tm* reached 60°C, no nonspecific amplification was observed and the test was shown to be highly sensitive compared with the other methods tested.

### Multiplex DPO PCR is sensitive and specific for the detection of influenza A and B

Template concentrations as low as 10^2^ viral particles/ml were detectable using this method, with a robust test result obtained by multiplex DPO PCR at a concentration of 10^3^ viral particles/ml when applied to a mixture of influenza virus templates. A previous study reported the sensitivity of 10^2^ copies per reaction for each type of virus; however, the concentration of the mixture of viruses amplified was not stated [5,6]. The DPO primer amplified PCR products for seasonal H1N1, 2009 pandemic H1N1, H3N2 and avian influenza H5N1 were clearly visualized by agarose gel electrophoresis without non-specific amplification or false-positive bands (Figure 3).

Test specificity was investigated in three ways. Each DPO primer pair was individually applied towards amplifying a pooled mixture of the five influenza viruses tested, and a single distinct band was produced for each template (Figure 4A). Secondly, a multiplex PCR primer pool was applied towards amplifying each target template individually, and yet again, no non-specific bands were detected (Figure 4B). Furthermore, no non-specific amplification was observed when applying the multiplex DPO PCR primers to other, non-targeted virus templates (Figure 4C). A previous study stated that the DPO system prevents non-specific amplification without inhibiting the efficient amplification of the target bands [11]. In this study, the high sensitivity and specificity were consistent with previous studies [5,6].

### Evaluation of DPO PCR performance to detect viruses in clinical specimens

This study evaluated the multiplex DPO PCR system and compared it with qPCR using 233 respiratory clinical specimens. All clinical samples were screened by singleplex qPCR for the separate detection of influenza A and B. Positive specimens were defined as those reaching a florescent threshold value (Cq < 35), and were used as standards. Based on the statistical results, the multiplex DPO PCR showed no significant difference in sensitivity or specificity with conventional single PCR, and had a higher sensitivity and specificity than the colloidal gold immunochromatographic assay.

Several previous studies have evaluated different respiratory virus diagnostic tests using the DPO-based commercial kit including Seeplex® RV15 ACE Detection kit and RV 12 Detection kit (Seegene). Their results indicated that the sensitivity and specificity of the Seeplex DPO based PCR system were 83.3% and 95.2%, respectively [12]. Cho *et al.* showed that the sensitivity and specificity of Seeplex RV15 DPO PCR for influenza A were 93% and 99.9%, respectively, and for influenza B were 80% and 99.9%, respectively [13]. Previous studies only used nasopharyngeal swabs or nasal washs, but not oropharyngeal swabs, as their main clinical sample types. The variation in sensitivity and specificity of influenza virus detection between this study and previous studies might be due to different patient sampling sites, which might result in different virus loads between the two sample panels. Commonly used clinical specimens for the detection of respiratory viruses are oropharyngeal swabs, nasopharyngeal swabs, nasal washees and sputum. However, different sampling methods may give rise to a change in the detection of influenza virus sensitivity. Li *et al.* indicated that nasopharyngeal swab samples may be the most effective alternative to nasal washes and oropharyngeal swab samples for the examination of respiratory viruses in adults [14,15]. Another study also suggested that the use of nasopharyngeal swabs was superior to oropharyngeal swabs for the detection of influenza viruses by PCR [16]. Indeed, the determination of the correct sampling site for collection of clinical specimens might be the most important factor affecting the successful detection of influenza viruses.

### Comparison between DPO PCR and qPCR, conventional single-target PCR and immunochromatographic assay

In this study, no significant difference in sensitivity was observed between DPO PCR and conventional PCR. Although the use of conventional primer has a low cost and requires less technical training for lab technicians, this approach often produces false positive results with low sensitivity and specificity. The use of conventional primers has a number of disadvantages including primer competition, primer dimers and varing anealing temperatures required for different primers. The current use of multiplex conventional PCR requires further validation including nested PCR or probe hybridization assay to verify the results [17].

In addition to the use of PCR methods, the rapid detection of influenza virus can also be carried out using the immune colloidal gold technique supplied as commercial kits. The current study demonstrated that this rapid detection method had the lowest sensitivity and specificity for virus detection when compared with the three different PCR based methods (Figure 8).

Although qPCR is usually used as a gold standard in most laboratories to detect influenza virus, the standard Cq value should be approximately 35 to determine a positive outcome as suggested by the World Health Organization [10]. However, the World Health Organization strongly suggests that the assay should be repeated to verify the diagnostic result if the Cq value is in the range 30–35 as determined by qPCR [10]. From the comparative study of positive specimens detected by qPCR and multiplex DPO PCR in this study, 89.3% of influenza A positive specimens were observed at a higher Cq range (30 < Cq < 35). The analytic results from influenza B testing indicated that 96.5% samples were in the Cq range from 30 to 35 (Figure 10). The high percentage of qPCR positive specimens not detected by multiplex DPO PCR might have had a low target concentration or were degraded by re-freezing of the sample [4].

### Limitations

The multiplex DPO PCR has some limitations. Most studies consider virus cultures and qPCR as a gold standard for influenza virus detection. In this study, virus culture was not available for use for all the influenza viruses detected.

## Conclusions

In conclusion, the optimized multiplex DPO PCR assay provides reliable sensitivity and specificity for the detection of seasonal H1N1, 2009 pandemic H1N1, H3N2, influenza B and avian influenza H5N1. Furthermore, the sensitivity of DPO PCR was higher than for the immunochromatographic assay, lower than for qPCR and similar to conventional single PCR for the detection of influenza A and B. Multiplex DPO PCR is cost-effective, has a short running time and low technical requirements indicating its significant potential for influenza virus detection, early diagnosis and treatment in clinical laboratories.
